# The association between continuous renal replacement therapy as treatment for sepsis-associated acute kidney injury and trend of lactate trajectory as risk factor of 28-day mortality in intensive care units

**DOI:** 10.1186/s12873-022-00589-6

**Published:** 2022-02-28

**Authors:** Zichen Wang, Luming Zhang, Fengshuo Xu, Didi Han, Jun Lyu

**Affiliations:** 1 Department of Intensive Care Unit, The First Affiliated Hospital of Jinan University, Guangzhou, Guangdong Province China; 2 Department of Public Health, University of California, Irvine, USA; 3 Department of Clinical Research, The First Affiliated Hospital of Jinan University, Guangzhou, Guangdong Province China; 4 School of Public Health, Xi’an Jiaotong University Health Science Center, Xi’an, Shaanxi Province China

**Keywords:** Continuous renal replacement therapy, Sepsis, Acute kidney injury, Lactate, Joint model

## Abstract

**Background:**

Sepsis has high incidence and fatality rates in intensive care units, often leading to renal failure. The effectiveness of continuous renal replacement therapy (CRRT) in sepsis-associated acute kidney injury (S-AKI) patients is currently uncertain.

**Aim:**

Joint model was used to determine the association between CRRT and the lactate trajectory trend and how it correlated to 28-day mortality for S-AKI patient in ICU.

**Methods:**

A retrospective study was applied to patients with sepsis and AKI, which were extracted from the MIMIC-III public database, with the endpoint being 28-day mortality. Every lactate level measurement within 28 days was observed and calculated using logarithms. Joint model combined the longitudinal analysis of the natural logarithm of the lactate level [log(lactate)] in longitudinal submodel and Cox regression by trajectory function, demonstrating the effects of CRRT on 28-day survival and log(lactate) changes, and its final relationship with the event status.

**Results:**

Among the 717 S-AKI patients, 157 received CRRT. CRRT was not associated with 28-day mortality. After adjustments, the relationship between CRRT use and log(lactate) elevation was statistically significant. The parameter estimation of CRRT and log(lactate) indicated that using CRRT will increase log(lactate) by 0.041 in S-AKI patients. The joint model also instigated a fixed association between changes in the lactate level and the event result, revealing an exp value of (0.755) = 2.12, indicating that an increase of one unit in log(lactate) will increase the risk of 28-day mortality 2.12-times.

**Conclusion:**

There was no significant association between CRRT use and 28-day survival in S-AKI patients, and JM showed that CRRT use might be associated with elevation of longitudinal lactate levels. Therefore, additional attention should be paid to other treatments to control lactate levels when providing renal support for patients with S-AKI.

**Supplementary Information:**

The online version contains supplementary material available at 10.1186/s12873-022-00589-6.

## Introduction

Sepsis is a common clinical critical illness [1] that causes dysfunction of the immune and blood coagulation systems, and affected patients usually have insufficient tissue perfusion [2]. As a product of glycolysis, lactate capacity directly reflects the degree of anaerobic glycolysis in tissues and cells, and is therefore a commonly used clinical indicator that effectively reflects tissue hypoxia and hypoperfusion. It has also been shown to be closely related to the prognosis of sepsis patients [3]. Several studies have shown that sepsis patients can have increased lactate levels due to low clearance or liver and kidney dysfunction [4].

Sepsis is also characterized by persistent refractory hypotension, hyperlactic acidemia, and organ dysfunction after aggressive fluid resuscitation [2]. Studies have indicated that sepsis can often lead to acute kidney injury (AKI). Up to 50% of sepsis patients develop AKI, leading to poor prognosis and a mortality rate (75%) [5, 6] that is significantly higher than that in sepsis patients without organ failure [7]. Continuous renal replacement therapy (CRRT) is a method of extracorporeal blood purification. Current clinical practices consider CRRT to able to maintain internal environment stability by removing toxic substances from the kidneys, and also supports organs function. CRRT has recently been widely used in sepsis treatment [7, 8]. However, the efficacy of CRRT in S-AKI patients remains uncertain. Studies have indicated that lactate levels and the 24-h lactate clearance rate after CRRT use are related to the 28-day mortality of S-AKI patients [9], while CRRT with massive hemofiltration is ineffective for severe lactic acidosis [10, 11].

A joint model (JM) technique was used in this study to investigate the influence of CRRT on lactate levels and its efficacy on S-AKI patient survival. Applying a JM to longitudinal and event time data has become a valuable follow-up data analysis method that combines the linear mixed model for longitudinal data and the Cox proportional-hazards model for time-to-event data by trajectory function [12]. A JM provides a more effective method for predicting the impact of treatments on outcomes, a more effective estimation of treatment effects on longitudinal data, and reduces the bias of the overall prediction when compared to the single linear mixed model and the Cox proportional-hazards model [12, 13].

## Methods

### Data source and extraction

Data were extracted from the Medical Information Mart for Intensive Care-III (MIMIC-III) database, which is a public database containing data on over 50,000 patients in critical-care units [14]. SQL was used to extract data before further analysis. The data extracted were the natural logarithm of the lactate level [log(lactate)], CRRT use, ventilator use, vasopressor use, demographic data including age, sex, and race, vital signs including mean blood pressure, respiration rate (RR), heart rate, skin temperature, oxygen saturation, and partial pressure of carbon dioxide, laboratory test results including urine output (UO), pH, albumin, bicarbonate, Serum Creatinine (sCr), glucose, magnesium (MG), phosphorus (PHOS), potassium, sodium (NA), hematocrit, hemoglobin, platelet, red blood cell width (RDW), white blood cells, neutrophil/lymphocyte ratio (NLR), comorbidities including heart failure, hypertension, liver diseases, fluid-electrolyte imbalance, and SOFA score.

### Study population

This study selected 1295 S-AKI patients from the MIMIC-III database. The inclusion criteria included meeting Sepsis-3 criteria [15], since they have severe infection and organ failure (SOFA score ≥ 2); AKI that occurred during hospitalization (increase in SCr by 0.3 mg/dl [6.5 µmol/l]) within 48 h; a 1.5-times increase in SCr from baseline, which is known or presumed to have occurred within the previous 7 days; urine volume < 0.5 ml/kg/hour for 6 hours [16]. Patients younger than 18 years(*n* = 3) and without longitudinal records of lactate levels within 28 days(*n* = 575) were excluded, leaving 717 patients as the study population.

### Statistical analysis

#### Longitudinal data analysis

Every lactate level measurement made within 28 days in this study was observed and assessed using logarithms. Linear mixed-effect models were used to analyze longitudinal data. The dependent variable was log(lactate). The independent variables were initial lactate level, CRRT use, time of lactate level observation (Time), and two-way interaction between CRRT and Time(CRRT*Time).

#### Time-to-event data analyses

Time-to-event data were first analyzed using the Cox proportional-hazards model to determine the relationship between CRRT and 28-day survival. Variables were screened using multivariable regression if they differed significantly between the CRRT and non-CRRT groups by univariable analyses.

#### Joint model

**Fig.1:**
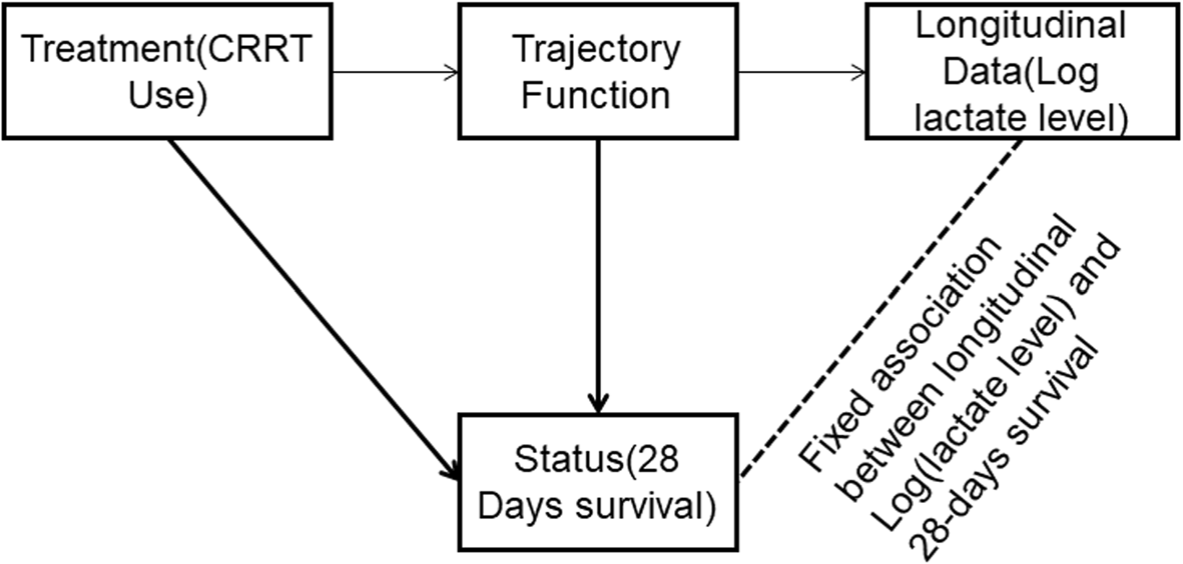
The Structure and Factors Relationship of Joint Model

The JM combined the longitudinal analysis of log(lactate) and Cox regression using a trajectory function, revealing the effect of CRRT on 28-day survival as well as log(lactate) changes and their relationship with the event status (Fig. 1).

All statistical analyses were performed using R software, with the JM being constructed using the “JM” package. Continuous variables were presented as medians with normality-based quartiles, with p values calculated using Student’s t-tests. Categorical variables were presented as numbers and percentages, with p values calculated using chi-square tests.

## Results

### Patient characteristics

Among the 717 S-AKI patients, 157 received CRRT. Table 1 lists the baseline characteristics of the two CRRT groups including demographics, vital signs, laboratory-result comorbidities, and SOFA scores. These characteristics demonstrate that CRRT was associated with significant differences in age, race, RDW, NA, MG, PHOS, CR, and UO, liver disease status, ventilator and vasopressor use, SOFA score, and 28-day mortality.

**Table 1:** Baseline characteristics between CRRT Unreceived /Received Group

Characteristics	CRRT Unreceived	CRRT Received	*p* value
N	560	157	
Age	71.0(61.0,81.0)	67.0(58.0, 77.0)	0.001^*^
Sex n,(%)			0.459
Male	347(62.0)	103(65.6)	
Female	213(38.0)	54(34.4)	
Ethnicity n,(%)			0.013^*^
White	408 (72.9)	109 (69.4)	
Black	85 (15.2)	16 (10.2)	
Others	67 (12.0)	32 (20.4)	
Vital signs			
Mean blood pressure (mmHg)	70.3(65.1,75.6)	68.4(64.1,74.6)	0.057
Heart rate (min-1)	84.0(73.9,98.2)	86.9(76.8,97.3)	0.271
Respiratory rate (min-1)	19.7(17.4,22.9)	19.9(17.4,22.8)	0.633
Oxygen saturation (%)	97.5(96.2,98.9)	97.2(96.0,98.7)	0.119
Skin temperature (℃)	36.7 (36.2, 37.2)	36.7 (36.2, 37.3)	0.906
Partial pressure of carbon dioxide (mmHg)	39.0(33.0,48.0)	42.0(36.0,48.0)	0.091
Laboratory result			
White Blood Cell (k/uL)	11.80 (7.40, 17.12)	11.70 (7.40, 16.80)	0.850
Neutrophil/lymphocyte ratio	11.86 (5.90, 21.80)	11.11 (6.80, 21.50)	0.563
Hemoglobin (g/dL)	10.60 (9.40, 12.10)	10.70 (9.30, 11.90)	0.731
Red blood Cell Distribution Width (%)	16.25 (14.80, 18.20)	16.80 (15.50, 18.40)	0.023^*^
Hematocrit (%)	32.60 (28.90, 37.00)	32.60 (28.50, 37.40)	0.999
Platelet (K/uL)	194.00 (126.25, 272.25)	196.00 (120.00, 271.00)	0.972
Sodium (mEq/L)	138.00 (134.00, 141.00)	137.00 (133.00, 140.00)	0.017^*^
Potassium (mEq/L)	4.50 (4.00, 5.20)	4.60 (4.00, 5.30)	0.320
Magnesium (mg/dL)	2.00 (1.70, 2.30)	2.00 (1.80, 2.40)	0.034^*^
Bicarbonate(mEq/L)	22.0(19.0,26.3)	22.0(18.0,26.0)	0.526
Phosphate(mg/dL)	3.90 (3.00, 5.00)	4.60 (3.50, 6.20)	< 0.001^*^
Albumin(mg/dL)	2.9(2.5,3.3)	2.8(2.4,3.3)	0.629
Glucose(mg/dL)	128.00 (102.00, 179.25)	126.00 (98.00, 175.00)	0.789
Creatinine(K/uL)	2.70 (1.87, 4.20)	3.70 (2.70, 5.20)	< 0.001^*^
PH	7.4(7.3,7.4)	7.3(7.3, 7.4)	0.061
Urine output (mL)	804.0 (258.5, 1459.4)	195.0(39.0, 602.0)	< 0.001^*^
Comorbidities, n (%)			
Congestive heart failure	310 (55.4)	91 (58.0)	0.624
Hypertension	448 (80.0)	121 (77.1)	0.490
Liver disease	123 (22.0)	47 (29.9)	0.049*
Fluid electrolyte	343 (61.3)	96 (61.1)	0.999
Ventilator use n,(%)	255	119	< 0.001^*^
No	305(45.5)	38(75.8)	
Yes	255(54.5)	119(24.2)	
Vasopressor use n,(%)	374	155	< 0.001^*^
No	186(33.2)	2(1.3)	
Yes	374(66.8)	155(98.7)	
SOFA	8.00 (6.00, 10.00)	11.00 (9.00, 12.00)	< 0.001^*^
28-Days Mortality n,(%)			0.009
No	307(54.8)	67(42.7)	
Yes	253(45.2)	90(57.3)	

**Fig.2:**
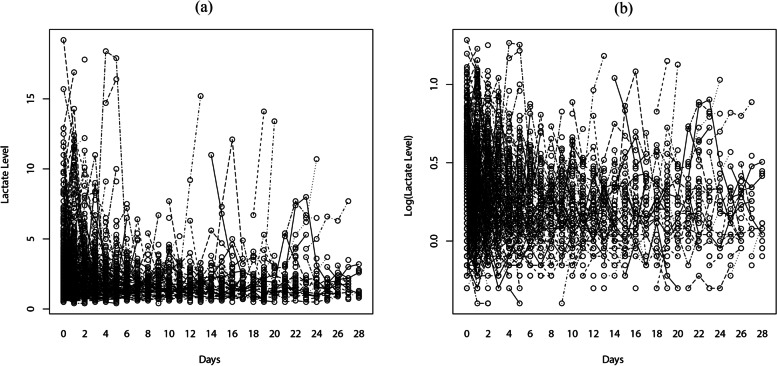
The trajectory record of 3661 lactate value (**a**) and Log(Lactate) value (**b**). Each line stands for a trajectory for one patient, there are 717 lines. **a** and **b** represents the lactate change of 717 patients involved in the study

**Fig.3:**
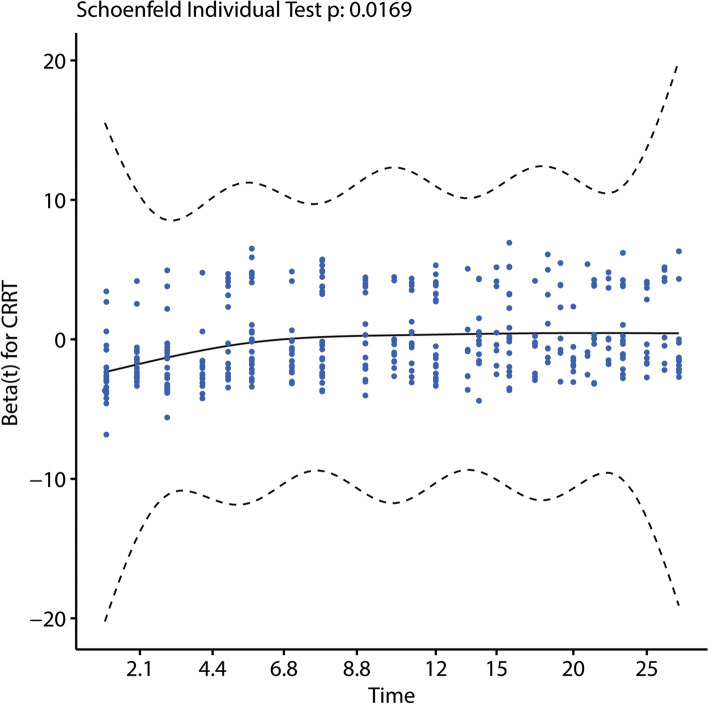
Schoenfeld Residuals Test result of CRRT in Cox regression analysis. The *p*-value of Schoenfeld Residuals Test result is larger than 0.05 which indicated that CRRT is not a time dependent variable and can be analyzed by Cox Proportional Hazards Model

The longitudinal lactate data of 3661 observations were displayed using trajectory functions and plotted using interaction figures. Figure 2 indicates the linear trajectory record of lactate and log(lactate) for each patient over the 28-day analysis period. The figure shows that most observations were concentrated within the first 5 days after patient admission. Lactate levels of patients ranged from 0.5 to 20, with log(lactate) ranging from –0.4 to 1.3. Cox regression was performed to analyze the relationship between CRRT and 28-day survival, with CRRT being adjusted for by age, race, RDW, NA, MG, PHOS, SCr, and UO, liver disease status, ventilator and vasopressor use, and SOFA score. The Schoenfeld residuals test (Fig. 3) was used to determine the independence of residuals and the time to test for the proportional-hazards hypothesis in the Cox model. The results in Fig. 3 revealed that CRRT was not a time-dependent variable and therefore could be analyzed directly using Cox regression. CRRT was also found to not be related to 28-day survival in S-AKI patients after adjusting multiple variables.

Linear mixed-effect models, on the other hand, indicated the association between CRRT and log(lactate) was not significant. Among the independent variables, the initial lactate level and Time were statistically correlated with log(lactate) in longitudinal status. The result of the JM demonstrated that after combining the longitudinal submodel and the survival model, CRRT was still not related to the 28-day survival of S-AKI patients while age, RDW, UO, and trajectories of log(lactate) were significantly associated with 28-day mortality in S-AKI patients (Supplementary Table 1). However, the fixed JM result revealed that the correlation between CRRT use and elevation of log(lactate) was statistically significant. The parameter estimation of CRRT and log(lactate) indicated that using CRRT increased log(lactate) by 0.017 in S-AKI patients. The JM also instigated a fixed association between lactate level changes and the event result, producing an exp value of (0.755) = 2.12, indicating that an increase of one unit of log(lactate) will increase the risk of 28-day mortality 2.12 times (Table 2).

**Table 2:** Result of effects between two submodels and the joint model

Parameter	COX			LME			JM		
	Parameter Estimate	SE	*P* value	Parameter Estimate	SE	*P* value	Parameter Estimate	SE	*p* value
CRRT Treatment Effect on 28-Days Survival	-0.167	0.123	0.178	/	/	/	0.182	0.141	0.196
CRRT Treatment Effect on Log(Lactate)	/	/	/	0.013	0.008	0.116	0.017	0.006	0.008^*^
Log(Lactate) Effect on 28-Days Survival	/	/	/	/	/	/	1.755	0.196	< 0.001^*^

*Cox *Cox Proportional Hazards Model, *LME *Linear Mixed Effect Model, *JM *Joint Model

LME submodel independents includes: CRRT, Time, CRRT*Time, initial lactate level

Cox submodel was adjusted by: Age, ethnicity, RDW, NA, MG, PHOS, CR, UO, liver disease status, ventilator use, vasopressor use, and SOFA score

^*^p-value is less than 0.05

## Discussion

Our survival submodel indicates that CRRT was not significantly associated to the 28-day mortality of S-AKI patients in critical care. S-AKI patient often have weaker glomerular filtration functions, which can increase the probability of adverse symptoms such as electrolyte/acidolysis imbalance and also impair metabolism [17]. Therefore, preventing further aggravation through eliminating metabolic toxins to prevent further aggravation is necessary. CRRT is currently one of the main treatment methods [18]. This intervention focuses on replacing kidney function, removing toxic substances such as CR, or improving the fluid-electrolyte imbalance that allows for future treatments since patients are in a stable condition [18–20]. Although CRRT is still the most common treatment for S-AKI, studies have indicated that renal replacement therapy cannot improve its survival [8, 21]. Whether to apply high-intensity CRRT to S-AKI patients remains controversial. Some studies have indicated that the use of high-dose renal replacement therapy does not improve the overall survival of patients or the recovery of renal function [21, 22]. The efficacy of CRRT on the prognosis of S-AKI can therefore not be confirmed.

The analysis of longitudinal submodel indicated that the use of CRRT tends to increase lactate levels within 28 days, while the joint modeling of longitudinal and survival data indicated that lactate level changes were associated with mortality and that log(lactate) is a risk factor for 28-day mortality in S-AKI patients. Many studies have confirmed lactate as a powerful biomarker for sepsis with renal damage and can accurately predict mortality [23–25]. Reducing lactate is therefore a vital procedure for improving the likelihood of patient survival. However, although CRRT is the most popular treatment in patients with sepsis and AKI, there is still a possibility that it cannot reduce acidosis, resulting in the continued elevation of lactate levels after CRRT is performed [26]. Potential explanations include how in clinical practice, since it is generally believed that if lactate fluctuates to a lower range, there will be no negative impact on the prognosis of patients. Therefore, these lower lactate levels may not receive adequate attention. Additionally, lactate levels may be affected by the buffer used in CRRT [27]. For example, when using lactate-based fluids, sepsis patients failed to completely metabolize lactate [28], potentially leading to increased lactate levels, eventually developing into metabolic acidosis hyperlactatemia. Finally, when sepsis patients with AKI have more serious infections, vascular permeability is increased and the responses to vasopressors are poor. Although CRRT can temporarily remove toxins and other substances from the body, circulatory ischemia and hypoxia still cannot be improved, and lactate will also continuously increase [29]. Our research found that across all ranges of lactate levels, the risk of death increased 2.12-times for every unit increase in log(lactate). When using CRRT to treat patients with sepsis and AKI, we should therefore pay more attention to changes in patient lactate levels.

This study had some limitations. Some factors during the use of CRRT will impact survival, such as filter coagulation, the conversion of patients to intermittent hemodialysis after hemodynamic stability is reached, and the death of patients during treatment. The limited database means we could not analyze these impacts retrospectively. Additionally, the single-center nature of the database reduces the generalizability of our results, which must therefore be tested in further research.

## Conclusion

There was no significant association between CRRT use and 28-day survival in S-AKI patients, and JM showed that CRRT use might be associated with elevation of longitudinal lactate levels. Therefore, additional attention should be paid to other treatments to control lactate levels when providing renal support for patients with S-AKI.

## Supplementary Information

**Additional file 1:** **Supplementary Table 1.** The full result of the Joint Model.

## Data Availability

The data were available on the MIMIC-III website at https://mimic.physionet.org/, https://doi.org/10.13026/C2HM2Q. The data in this article can be reasonably applied to the corresponding author.
